# A Prospective Review of the Sensory Properties of Plant-Based Dairy and Meat Alternatives with a Focus on Texture

**DOI:** 10.3390/foods12081709

**Published:** 2023-04-20

**Authors:** Rachael Moss, Jeanne LeBlanc, Mackenzie Gorman, Christopher Ritchie, Lisa Duizer, Matthew B. McSweeney

**Affiliations:** 1 School of Nutrition and Dietetics, Acadia University, Wolfville, NS B4P 2K5, Canada; 2 Department of Food Science, University of Guelph, Guelph, ON NQG 2W1, Canada

**Keywords:** plant-based foods, dairy analogues, meat analogues, texture, oral processing

## Abstract

Consumers are interested in plant-based alternatives (PBAs) to dairy and meat products, and as such, the food industry is responding by developing a variety of different plant-based food items. For these products to be successful, their textural properties must be acceptable to consumers. These textural properties need to be thoroughly investigated using different sensory methodologies to ensure consumer satisfaction. This review paper aims to summarize the various textural properties of PBAs, as well as to discuss the sensory methodologies that can be used in future studies of PBAs. PBAs to meat have been formulated using a variety of production technologies, but these products still have textural properties that differ from animal-based products. Most dairy and meat alternatives attempt to mimic their conventional counterparts, yet sensory trials rarely compare the PBAs to their meat or dairy counterparts. While most studies rely on consumers to investigate the acceptability of their products’ textural properties, future studies should include dynamic sensory methodologies, and attribute diagnostics questions to help product developers characterize the key sensory properties of their products. Studies should also indicate whether the product is meant to mimic a conventional product and should define the target consumer segment (ex. flexitarian, vegan) for the product. The importance of textural properties to PBAs is repeatedly mentioned in the literature and thus should be thoroughly investigated using robust sensory methodologies.

## 1. Introduction

There has been significant growth in food demand due to the growing global population. With this growth comes a shift in the types of foods that consumers are looking for, and plant-based products have gained considerable attention. This increased demand for plant-based products may be a result of the rapidly growing trends of veganism and vegetarianism, as well as the increased consumption of plant-based food in Western countries. In the United States, the prevalence of vegan diets has increased by 500% from 4 to 19.6 million between 2014 and 2017 [1]. The consumption of plant-based foods is frequently encouraged to help reduce the negative impacts associated with the modern food supply, as well as to improve human and global health [1,2]. The growing consumer interest in reducing dietary animal-based products can also be attributed to sustainability and ethical reasons [1,3,4,5].

In response to the increasing consumer demand for plant-based products, the food industry is creating plant-based alternatives (PBAs) for meat, fish, cheese, milk, and yogurt. Among all the plant-based food products in the United States, plant-based milk or beverages (PBBs) were shown to have the highest market value, at USD 2,016,540, while plant-based meat analogues (PBMAs) followed at USD 939,459 in 2019 [6]. Moreover, PBBs experienced a growth of 14% in the United States market over a two-year period (from 2017 to 2019) and PBMAs had a 38% market share growth within this same period [2]. In Canada, dairy milk consumption decreased from 70.2% to 56.1% between 2004 and 2015, while PBB consumption increased from 1.8% to 3.0% [7].

Products made from plant-based ingredients aim to mimic the nutritional components and sensory properties of animal-based foods [3,8]. As such, the careful selection of ingredients is necessary to optimize the texture and flavor, and to ensure the adequate binding, color attributes, and nutrition of PBAs [9]. The challenge with PBAs is creating a product that has an acceptable appearance, texture, and flavor, all while maintaining functionality and nutrient integrity using affordable and sustainable plant-based ingredients. An additional hindrance to the creation of quality PBAs is the molecular and physiochemical differences between plant-based and animal-derived foods and ingredients [10]. These factors make it difficult to create PBAs that can completely replace their animal-based counterparts and be accepted by consumers. Studies have found that plant-based dairy alternatives, for example, are unable to completely replace cow’s milk in terms of its nutritional profile and sensory properties [3,11,12,13]. Many plant-based alternatives possess off-flavors, and are described as bitter and astringent [14]. In addition, plant-based alternatives have a lower amount of proteins, minerals, amino acids, and vitamins compared to cow’s milk [3].

Plant-based milk or beverages (PBBs) have become popular in recent years and are widely available in the market. This may be due to the frequent occurrence of dairy allergies, lactose intolerance, caloric concerns, as well as the increased prevalence of high cholesterol [15]. In addition to PBBs, there are also a variety of products that mimic animal-derived cheese, fermented dairy products, kefir, ice cream, and yogurt [16,17,18,19]. Plant-based alternatives to meat (PBMAs) are also being developed as a high consumption of meat has been shown to contribute to negative health consequences. Adverse health risks and an increased prevalence of cardiovascular disease and cancer have been associated with high red meat and processed meat consumption [20,21], while lower body weight, blood pressure, and cholesterol levels have been associated with plant-based protein consumption [22]. Environmental concerns also exist. Livestock produce high global greenhouse gas emissions and require significant feed to yield a small amount of meat for human consumption. Along with environmental and human health concerns, a shortage of meat supply is also anticipated in the future due to population growth and high meat consumption in humans. As a result, health authorities and organizations have emphasized the need to reduce the consumption of animal-based products and incorporate more plant-based foods [23]. However, plant-based foods may also pose some risks to consumers as they may contain allergens, thermally induced carcinogens, and anti-nutrients [24].

In response to the aforementioned factors, in addition to the emphasis on consuming more plant-based foods, food system transformation is occurring at a rapid pace. Technological strategies for manufacturing meat-like products from plant-based foods are growing rapidly and becoming readily accessible in markets worldwide [25]. The primary challenges that PBAs face revolve around consumer preferences, the products’ sensory properties, and the successful replication of meat products’ textural properties. A further challenge is the negative attitudes toward PBAs in comparison to conventional products [26,27]. This is largely related to the differences in taste and texture between conventional products and plant-based alternatives, further indicating the need for proper sensory evaluation studies to be conducted [27]. It is evident that the production of plant-based dairy and meat alternatives that mimic conventional products, particularly their textural properties, is a key challenge for product developers, and more research related to the relationships between the structural characteristics and specific sensory properties is necessary. The purpose of this review is to examine the sensory evaluation methods used in the assessment of plant-based dairy and meat products, with a focus on texture. The body of this review has two objectives:

1. To examine the sensory properties of plant-based meat and dairy alternatives with a focus on texture.
2. To discuss the sensory evaluation methodologies currently being used and make recommendations for methodologies that can be utilized in future studies for the evaluation of the sensory properties of plant-based alternatives.

## 2. Textural Properties of Plant-Based Alternatives

### 2.1. Meat Alternatives

The development of meat and fish alternatives has increased in recent years, leading to an increasingly diverse range of available products. Burger patties, fish and seafood products, nuggets, muscle-type products such as chicken or steak, minced products, as well as emulsion-based products, such as sausages, are now available [9,27,28,29,30]. Within these products, consumers are looking for textures, such as juiciness, tenderness, hardness, and chewiness, that are similar to their meat-based counterparts [31,32]. An example of the different studies that have been conducted on the textural properties can be seen in Table 1. Formulating products with these textures requires the appropriate selection of ingredients and a texturizing process that will lead to the acceptable sensory properties within the products.

#### 2.1.1. Ingredients

From an ingredient perspective, soy is one of the most commonly used ingredients in alternative meats. However, a number of ingredients, including textured vegetable protein (TVP), tempeh, tofu, algae, pulses, mushrooms, zein, seitan, nuts, beans, wheat, and seeds, are also commonly incorporated into meat analogue formulations [44]. PBMAs usually consist of proteins (gluten, soy protein, pea proteins, potatoes), binders (soy protein isolate, methylcellulose, carrageenan), fats (sunflower oil, canola oil, coconut oil, palm oil), and others (colorants and spices) [45]. The choice of ingredients is important for the consumers’ liking of the product. Yuan et al. [46] showed that it is possible to produce an acceptable analogue sausage through the incorporation of ground mushrooms with soybean protein isolate. However, Ettinger et al. [47] were not able to find an alternative protein suitable (the study included nuggets made from wheat flour, wheat gluten, rice flour, soy protein isolate, pea protein concentrate, textured soy protein) to produce a PBMA to chicken nuggets that is acceptable to consumers. It is often stated that texturized vegetable protein (TVP) exhibits a fibrous texture similar to traditional meat [33]. However, when compared to beef, pork, and chicken, overall, the textural and chemical properties of TVP were found to be similar only to chicken, with significant differences noted between TVP and beef [30,31]. The instrumental hardness, chewiness, and gumminess, as well as the evaluations of firmness and tenderness, were significantly higher in beef patties compared to the plant-based meat analogue, suggesting that significant textural differences exist between the products [31].

In addition to the type of alternative protein, the amount added into the product is important. Pea protein has been proposed as an ingredient that can increase the acceptability of the textural properties of PBMAs; however, there is a limit to the amount that can be used. Consumer testing has shown that as the amount of pea protein in a PBMA increases, the acceptability of the texture decreases [34]. The challenge with these plant-based protein sources is that they consist primarily of globular proteins that are not capable of forming the structure of traditional meat; for this reason, plant proteins and their additive ingredients often require intensive processing to achieve a texture that is accepted by consumers.

Fat also plays an essential role in the sensory properties of PBMAs by contributing to the mouthfeel and perception of juiciness. The characteristic marbling appearance of traditional meat can be achieved in plant analogues by whipping a mixture of oils into smaller globules of fat [37]. The fats from coconut and vegetable oils, such as rapeseed and sunflower, are typically used in meat analogues [48,49]. Vegetable oils, however, lack the meat-specific volatile compounds and texture, making it difficult to use for replicating the flavor and texture of meat [50]. Coconut oil or cocoa butter are better able to enhance the flavor and texture of the products [51]. There are many different fats that can be used in PBMAs, and the choice of fat to be used in the PBMA should be based on the processing method, type of product, and desired sensory quality. To discuss them all is beyond the scope of this review, however, an overview of the different fats used in PBMA production has been published by Kyriakopoulo et al. [45].

The juiciness in meat products is due to the immobilization of water by myofibrillar proteins, while in meat analogues, this function is achieved with the use of carbohydrate polymers, including plant fibers, starches, and polysaccharides (also called gelling agents) [52]. The addition of carbohydrate polymers also improves the texture by binding water and reducing syneresis [53]. Szpicer et al. [54] used konjac gum, carrageenan, xanthan gum, pectin, potato starch, and rapeseed oil in their PMBA formulation. Sixty untrained panelists evaluated the acceptability of the texture and juiciness of the PBMAs. The results identified that the textural properties are directly related to the gelling agents used in the formulation. The juiciness is directly impacted by the fat used in the PBMA, which can be liquid or solid, as well as emulsified. The emulsified fats are solid when the PBMAs are at room temperature, but are liquid when the product is heated [45]. Gelling agents also play a role during the cooking process, as they hold onto the water during cooking and increase the perception of juiciness in the PBMA [35].

#### 2.1.2. Processing Methods

During the development of PBMAs, the ingredients undergo a texturizing process to develop the appropriate texture. A number of different texturizing processes exist, including wetspinning, electrospinning, extrusion, creating a mixture of proteins and hydrocolloids, freeze structuring, shear cell technology, or 3D printing [44]. Structuring methods, such as extrusion, spinning, and shear cell techniques, have been widely studied and employed for texturizing vegetable proteins from pulses, grains, and oilseeds, while fermentation has been used for the growth of mycoproteins [27]. Among all the methods, extrusion and electrospinning are the most commonly used methods in the food industry for the production of PBMAs [9,52,55]. Extrusion subjects protein-based ingredients to hydration, high pressure, high temperature, and mechanical interactions through three steps. First, the proteins are mixed with water in an extruder, followed by cooking in a chamber with high pressure and high temperature, and lastly, the product is cooled in a cooling matrix [24]. To achieve the desired texture through extrusion, the water content, screw speed, matrix geometry, presence of polysaccharides, type of raw material, as well as processing temperature, all play a key role [56]. Rehrah et al. [57] used extrusion to create a peanut-based PBMA to beef patties and asked 60 consumers to evaluate their liking (using a nine-point hedonic scale) of the texture. The consumers scored their liking of the PBMAs quite low (less than five, relating to the dislike categories of the hedonic scale), except for one formulation, indicating that more work needs to be conducted to create an acceptable texture using peanuts and extrusion. Novel textures can also be created from proteins through wetspinning or electrospinning [52,58]. The benefits of wetspinning or electrospinning on the textural properties of PBMAs needs to be further explored using rigorous sensory evaluation techniques (e.g., quantitative descriptive analysis), as the studies conducted thus far have mainly used rheological parameters and texture analyzers.

Even with a wide variety of methods and applications to alter the textural properties, formulating products that accurately and successfully replicate traditional meat products remains a key challenge [25]. The texture of meat is highly valued by consumers, and is also crucial to consumer acceptance. Meat has a complex structure as a result of its natural texture, which is formed through muscle fibers, connective tissue, as well as other structural muscle elements, making it difficult to recreate from non-animal protein sources [59]. As such, several consumer studies have shown that the acceptance of meat analogues’ texture remains unsatisfactory. While attitudes and beliefs towards meat analogues, along with food neophobia, play a role in the acceptance of such products, the similarity to traditional meat (i.e., flavor and texture) is important to consumer acceptance [45]. Moreover, textural replication remains a key challenge for meat analogues, and thereby, for overall consumer acceptance. The type of structure that is achieved for a meat analogue is also highly dependent on the functional properties of the meat analogue ingredients themselves [55]. Therefore, it is crucial to understand the role and functional properties of each ingredient used in the product formulation to determine the texture outcomes for specific meat analogue product categories, such as fish or muscle meat [55].

To date, challenges exist with regard to mimicking the textural properties of meat, including the fibrous nature, mouthfeel, chewiness, tenderness, and cohesiveness. There is a particular problem with muscle-like tissue (sometimes defined as highly organized fine texture) [60]. Challenges exist with producing the appropriate juiciness perception due to the poor water binding capacity within the PBMAs. This leads to products being described as dry [28] or having an unacceptable mouthfeel [37].

### 2.2. Dairy Alternatives

#### 2.2.1. Plant-Based Beverages

There is strong interest in substitutes for dairy products. Plant-based beverages (PBBs) present a similar visual appearance to animal milk; however, they may present different mouthfeel and textural properties. PBBs are a colloidal system formed by a continuous phase of water and a dispersed phase of particles [61]. These particles consist of starch granules, plant matrices, protein fractions, and lipid droplets [62], and can lead to unappealing mouthfeels such as chalkiness and grittiness [63]. Different PBBs have different viscosities, and this can influence consumer acceptability [64,65] For instance, tree-nut-based PBBs have been designed, but their rheological properties are considered to be poor when compared to animal milks or soy-based PBBs [65]. In addition, some different ingredients in PBBs may lead to sedimentation, which is not acceptable to consumers [66].

Consumers are interested in the textural properties of PBBs, as evidenced by the word association task conducted with 323 Canadian participants in which mouthfeel properties (watery, creamy, thick and powdery) were consistently mentioned by the participants [14]. In the same study, six PBBs (soy, almond, oat, coconut, cashew, and pea) were evaluated for their sensory perception by consumers using hedonic scales and check-all-that-apply (CATA). The mouthfeel of the pea and almond PPBs was found to be liked significantly more than the coconut and cashew PBBs. Additionally, when a penalty lift analysis (combining overall liking and CATA) was conducted, it identified that creamy and smooth textures positively impacted the consumer liking, while a watery texture decreased the liking. However, in this study, the PBBs were not compared to dairy milk. Chickpea extract and coconut extract created a beverage that was comparable to dairy milk [13], but similar to the studies above, this study did not include a comparison to dairy milk. Furthermore, peanut milk was found to have a significantly higher mouthfeel liking than soy milk [67], but once again, the study did not include dairy milk. For food product developers to create a PBB that has a similar texture to dairy milk and is well-liked by consumers, sensory studies need to include a comparison of PBBs to dairy milk.

PBBs are subjected to ultra-high temperature treatment before being packaged in order to increase the shelf life; however, this processing can promote the degradation of thermolabile compounds and impact the textural properties of the beverage [68]. PBBs can also contain particle aggregates that cause a grainy or gritty mouthfeel, which reduces consumer acceptability [66]. Many PBBs have added sugar and salt that improves the texture, flavor, and shelf-life of the product [69,70]. Starches such as locust bean gum, pectin, and inulin can be added to PBBs as a thickening agent to improve the textural properties [19,66]. Fermentation is another method that could be used to improve the textural properties of PBBs. Black chickpeas were subjected to lactic acid bacteria (LAB) fermentation, and the resulting beverage was found to possess an improved consistency, cohesivity, and viscosity compared to the control beverage (without fermentation) [39].

PBBs are added to a variety of different food products, including coffee. Dairy milk has better foaming properties than PBBs at 4 °C when included in a cappuccino beverage; however, the properties were similar when compared at 65 °C [71]. This difference in the foaming properties may be because plant-based proteins are mainly globular in shape, and they will unfold upon mechanical stirring. This leads to the hydrophobic sections of the protein being exposed and absorbed at the air-liquid interface of the air bubbles [72]. Protein solubility influences the textural properties of foods, which needs to be considered during the production of plant-based beverages [73]. Overall, the textures of the soy, oat, and coconut/soy PBBs were significantly less liked by consumers at both temperatures than the dairy milk [71]. Others have found that the mouthfeel of oat, soy, and almond PBBs in coffee was not significantly different than that of dairy milk; however, the coffee with dairy milk scored highest for overall liking and was liked significantly more than the coffee with oat PBB, suggesting that something other than mouthfeel contributed to the score of consumer liking [74].

#### 2.2.2. Other Dairy Alternatives: Ice Cream, Yogurt, and Cheese

PBBs can also be used to produce alternatives to ice cream or frozen desserts, yogurts, and cheeses. Researchers have used a variety of different plant-based ingredients to create these food items. For instance, hemp, almond, pectin, and psyllium fibre were used to create ice cream. The researchers concluded that hemp and pectin can create a product with an acceptable consistency [17]. Walnut milk has also been used to create ice cream that was comparable to dairy-based ice cream; however, the study only included eight panelists [75]. Coconut-based ice cream was identified to have a finer texture than conventional ice cream [76]. Similar to the PBBs, the main ingredient directly impacts the textural properties of the ice cream, and some of the formulations include different body agents and thickeners to improve the texture [77].

Plant-based yogurts (PBYs) must also achieve similar textural properties to conventional yogurt, including the proper viscosity, adherence to the spoon, and organoleptic perception, to be acceptable to consumers [18]. A dynamic sensory analysis method demonstrated that the typical properties used to describe dairy-based yogurts are also applicable to PBYs [78]. The main driver of consumer liking is thickness and creaminess, while wateriness and thinness decrease the liking [79]. Currently, PBYs, as identified by Giacalone et al. [80], tend to have different textural properties than conventional yogurt due to the lower protein concentrations and different gelation properties of the proteins compared to caseins. A study found that soy- and coconut-based yogurts had textures similar to that of conventional yogurts [81]. Additionally, quinoa- and soy-based yogurts were found to have acceptable textures to consumers; however, the study only included 15 participants [82]. Many PBYs usually use additives such as protein extracts, inulin, thickeners, and emulsifiers to create acceptable textures [83]. Coconut-milk-based PBY made with the addition of tapioca starch was found to improve consumers’ liking of the texture (nine-point hedonic scale) and the authors indicated that the consumers preferred the firmer textured yogurt [40].

Soy- and coconut-based PBYs currently dominate the market [84]. Grasso et al. [81] had 25 consumers evaluate five different commercially available PBYs and conventional dairy yogurt. They found that one of the soy yogurts and the coconut yogurt were not significantly different in terms of the consumers’ liking of the yogurts; however, most of the participants (36%) still preferred the dairy yogurt over all of the PBYs. Soy-based PBY was also investigated by Xu et al. [41]. They added hemp to the soy PBY and found that a 10% addition level led to an increase in the liking of the texture by the participants (n = 20) when compared to the control without hemp addition. Soy was also mixed with quinoa and then underwent LAB fermentation to produce another PBY [82]. The participants’ (n = 15) liking of the texture was significantly increased by the addition of texture. Lentils and other pulses have begun to be used in the production of PBYs. A preliminary sensory investigation (n = 13) found that PBY made from red lentils had acceptable firmness, however, the PBYs were not compared to conventional yogurt. However, both of these studies used a small number of consumers, and future studies should use 50 or more consumers to evaluate the acceptability [85].

The issue with textural properties continues when investigating plant-based cheeses (PBCs), as soy-based cheeses have been found to have a gritty mouthfeel and grainy texture [86], as well as lower meltability than dairy cheese [87]. PBCs, similarly to dairy cheese, are emulsions of oil-in-water as the protein functions as an emulsifier and provides structure throughout a gel matrix [88]. PBCs have lower hardness compared to conventional cheese and they are less elastic and stretchy. Many PBCs are made from soy [89], and the coagulant used in the production of PBCs can also influence the texture; three soft soy PBCs were created with lime juice, steep water, and alum, and the liking of the mouthfeel was found to be significantly different across the 20 participants [90]. The lime-coagulated PBC’s mouthfeel was liked significantly more than the PBC made with steep water. A spreadable soy-based PBC was also compared to a PBC made with a 70:30 ratio of soy milk to almond milk, and although the participants’ (n = 50) liking of the PBCs on the nine-point hedonic scales was not significantly different for the texture, the overall acceptability was significantly higher for the soy- and almond-milk-blended cheese [91]. Spreadable PBCs have been fermented, and similar to PBBs, LAB fermentation has been used as a technique to reduce the gritty mouthfeel during the production of PBCs, as well as to achieve a smoother texture [92]. The fermentation of soybeans, followed by blending it with coconut milk (50:50 ratio), was found to significantly increase the participants’ liking of the texture compared to the 100% soy milk control; however, the trial only included ten participants [93]. Furthermore, using an emulsion-filled gel composed of olive oil and inulin with a dry-fractioned pea protein led to spreadable PBCs that had consistent textural properties, as assessed by trained panelists (n = 13) [94].

Although many PBCs are made with soy, coconut oil is also commonly used. Saraco and Blaxland [87] identified that among the 109 commercially available PBCs available in the UK, 74% had coconut oil as their primary ingredient. Based on this finding, the researchers evaluated four different coconut-oil-based PBCs; two were styled as mild cheddar and the others as semi-hard Italian. The panelists (one semi-trained and three trained panelists) determined that the semi-hard-Italian-styled PBCs had an unacceptable texture, and one mild-cheddar-styled PBC had an acceptable texture (but unacceptable flavors). The other mild-cheddar-styled PBC did not have a typical cheese texture, but it was deemed to have an acceptable texture. However, this cheese could be off-putting for consumers looking for a product that mimics dairy-based cheese.

A key property of cheese is its melting behavior. A study investigated the consumer acceptability of five commercially available PBCs in both raw and melted forms [95]. The PBCs were evaluated using hedonic scales and CATA. In addition, the participants were asked to answer an open-ended comment question about PBCs. In the open-ended comment question, the participants were concerned about the textural properties of PBCs and stated that PBCs tend to be rubbery. Overall, the five raw PBCs (made from different ingredients) scored very poorly for the liking of the texture, and only one PBC’s (made from modified potato starch and modified corn starch) average liking was above five on the nine-point hedonic scale (evaluated by 100 participants). The importance of the textural properties of PBCs was reinforced as a soft texture increased the participants’ liking, while a rubbery attribute decreased the liking (based on a penalty lift analysis). The PBCs were then evaluated in melted form (n = 93) and, overall, the melting decreased the participants’ liking of the texture. The participants’ liking was again influenced by the textural properties as smooth, creamy, and soft textures increased the liking, while mouthcoating and a rubbery texture decreased the liking. Overall, the melting behavior of the PBCs was not acceptable to the participants.

In general, the current dairy alternatives have textures that are unfamiliar to consumers, making them unacceptable. This includes gritty mouthfeels in PBBs and PBCs, as well as unacceptable textures in PBYs (due to different gelation properties). In addition, the functionality of dairy alternatives (addition of PBBs to coffee) and the melting behavior of PBCs further identifies that the textural properties of dairy alternatives are different from conventional dairy products.

## 3. Review of Sensory Evaluation Methods Used and Future Considerations

The sensory evaluation of any new food generally involves the use of consumer testing to examine the acceptability of the product. Sensory quality has been identified as the main barrier to the consumption of PBAs [96], and this may be due to consumers’ unfamiliarity with the products. In addition, PBAs are marketed with reference to their animal counterparts, and if these expectations are not met, they can lead to consumer dissatisfaction [80]. As shown in Table 2, consumer testing is the most common technique used by researchers when examining PBAs. The majority of the studies involved untrained panelists or consumers who were asked about their liking using affective tests and hedonic scales. These scales are used to assess the degree of product acceptance and usually consist of nine categories ranging between “dislike extremely” and “like extremely” [97]. The scales usually have a neutral category (“neither like nor dislike”) in the middle of the scale. These scales have been used extensively in the development of new food products [98], and as such, have been used to evaluate the acceptability of PBAs.

Many studies have evaluated the acceptability of the flavor and textural properties of PBAs. The sensory properties of PBAs are the most important driver of individual food choices [99]. The studies outlined in Table 2, using hedonic scales, mainly asked the panelists to evaluate the acceptability of the flavor and texture, as well as their overall liking. This may be a limitation of these studies as consumers have been found to mainly focus on the taste (understood as flavor by consumers) sensory modality when completing the hedonic scales [100]. Furthermore, flavors are directly related to consumers’ overall liking [101,102]. However, the flavor cannot be completely ignored when investigating the textural properties as there is an interaction between texture and flavor in food products. For instance, a fruity aroma has been found to decrease the thickness perception in low-fat yogurts [103]. Taste is also correlated to texture perception [104,105]. There are few studies investigating this cross-modality in PBAs; however, a study on PBYs found that lemon and vanilla aromas impacted the participants’ perceived sweetness, but did not impact the mouthfeel properties [79]. In addition, texture is a multidimensional sensory attribute that is difficult for consumers to understand, and individuals can have different frames of reference for texture, which can make it difficult for consumers to evaluate [106]. Future studies need to continue to investigate texture-related cross-modalities.

When conducting sensory tests, it is important to know if the developed product is intended to mimic a conventional product or if it is a new plant-based product with unique features [89]. By understanding the purpose of the study, proper treatments can be identified and examined. If the product is meant to mimic a conventional product, consumers will have expectations of the textural properties of these products [107], and if these expectations are not met, the PBAs will not be acceptable to consumers [108]. It may also be necessary to consider the addition of conventional products as comparators during testing. In the literature, few studies include a conventional product. However, if the objective of the work is to develop a PBA that is similar to its conventional counterpart, this treatment should also be included in the testing.

Careful thought should also go into the scales being used during sensory testing. Most studies only focus on the acceptability of the texture of the product as this is the main concern for the researchers. However, attribute diagnostic questions (ex. just-about-right scales (JAR), intensity scales) or check-all-that-apply (CATA) questions where panelists identify the properties perceived in the product, when combined with hedonic scales, will provide a greater depth of information about the PBA. JAR scales are one of the simplest consumer-based techniques to determine the optimum intensity of sensory properties [109]. A study on a PBA to salami asked participants to judge the hardness (too soft to too firm) and mouthfeel (three scales ranging between too dry and too watery, too tallowy and too oily, too coarse and too smooth) on JAR scales, with the middle of the scale representing just-about-right. Hedonic scales were also included [37]. The salami PBAs were formulated with soy protein isolate and the fat consisted of canola oil and differing amounts of sal fat (*Shorea robusta* seed oil) (0%, 25%, 50%, 75%, 100%). During the sensory trial, the participants were asked to not think about a conventional salami as their reference, but rather to base their expectations on an ideal plant-based salami replacement. The JAR scales were able to identify that the formulation with the 25% sal fat had the best textural properties.

Intensity questions have been recommended for product optimization [110], however, intensity scales may also be difficult for consumers if there is a large number of scales. Intensity scales are usually used by trained panelists and then combined with consumer liking scores using preference mapping. Preference mapping allows the researchers to determine which properties correlate with consumers’ or different consumers segments’ liking. A good example of preference mapping and PBAs can be found in the study by Lawrence et al. [111].

Check-all-that-apply (CATA) questions are simple for consumers to understand and are able to effectively characterize food products [112]. When CATA is combined with hedonic scales, a penalty lift analysis can be conducted to determine which properties significantly impact consumer liking [113]. CATA scales and hedonic scales were used to evaluate six PBBs [14]. The results of the CATA question and the nine-point hedonic scales were combined using penalty lift analysis, and the results identified that sweet, creamy, smooth, nutty, and white properties increased the consumer liking, while aftertaste, brown, beany, watery and off-flavor properties detracted from the liking. Similarly, rate-all-that-apply (RATA) was used to evaluate PBBs [114]. RATA asks the participants to select the sensory properties that apply to the current sample and then rate the intensity of the sensory property [115]. Although the results of the study mainly focused on the flavor of the PBBs, the RATA could be specifically applied to evaluate the textural properties of PBBs. By using the attribute diagnostic scales, the results generated will help product developers develop new PBAs by indicating what drives consumers’ liking of the PBAs.

The type of panelist used in the study is important. Many studies included participants who consume meat or dairy products. However, different consumer groups have different emotional responses to PBAs [116], and vegetarians may have feelings of disgust towards meat-like-textures [117]. Consumers should also be segmented based on their attitudes towards health and sustainability, as they have been found to impact the acceptance of PBAs [118]. In addition, consumers with higher income and educational levels, as well as younger age, have been found to be more accepting of PBAs [119,120,121]. These different consumer profiles need to be considered when conducting sensory testing on PBAs, as they may have a direct effect on the textural perception. Furthermore, there may be two distinct categories of consumers for PBAs, those that want products that mimic the texture of meat and dairy and those that do not, and identifying the appropriate consumer for the product is important for the accuracy of the results. The size of the recruited panel must also be considered. Many of the published studies use less than 50 participants, whereas it is recommended that consumer studies be conducted with between 75–150 untrained participants [122]. Larger sample sizes would allow for the segmentation of the population to determine the different consumer groups that may exist. A good example of a segmentation study on PBAs is that conducted by Cardello et al. [123], and similar studies to this should be conducted by specifically investigating consumers’ textural preferences for these products.

**Table 2:** Examples of the different studies investigating sensory properties of plant-based alternatives.

Type of Plant-Based Alternative	Sensory Method	Panelists	Reference
Plant-based analogues to chicken nuggets (compared to chicken control)	Consumer acceptability (hedonic scales and CATA)	105 untrained panelists	Ettinger et al. [47]
Plant-based analogues to beef patty (with beef control)	Trained Panel	10 trained panelists	Bakhsh et al. [124]
Peanut-based analogues to a beef patty	Consumer Acceptability (hedonic and JAR scales)	60 untrained panelists	Rehrah et al. [57]
Mushroom-based sausage analogue	Consumer Acceptability (hedonic and intensity scales)	32 untrained panelists	Yuan et al. [46]
Gluten-free and soy-free plant-based meat analogues	Consumer Acceptability (hedonic scales)	60 untrained panelists	Szpicer et al. [54]
Meat substitutes	Consumer Acceptability (hedonic scale)	93 untrained panelists	Elzerman et al. [125]
Beef, plant-based, and hybrid burgers	Consumer Acceptability (under blinded, expected, and informed conditions using hedonic scales and CATA)	99 untrained panelists	Grasso et al. [28]
Plant-based to chicken and beef	Consumer Acceptability (hedonic scales)	71 untrained panelists	Godschalk-Broers et al. [32]
Plant-based chicken sausage analogues and hybrid chicken sausages	Hedonic Scales	8 trained panelists	Kamani et al. [30]
Plant-based nugget	Consumer Preference (ranking scale)	42 untrained panelists	Yuliarti et al. [34]
Plant-based beverages (two trials: unflavored and flavored)	Consumer Acceptability (hedonic scales and CATA)	88 untrained panelists and 80 untrained panelists	Moss et al. [14]
Chickpea and coconut plant-based beverages	Consumer Acceptability (hedonic scales)	128 untrained panelists	Rincon et al. [13]
Dairy milk and plant-based beverages added to cappuccino	Consumer Acceptability (hedonic scales	50 untrained panelists	Zakidou et al. [71]
Plant-based alternatives to ice cream	Consumer Acceptability (hedonic scales)	30 untrained panelists	Leahu et al. [17]
Plant-based alternatives to yogurt (including two dairy controls)	Temporal Dominance of Sensations (mouthfeel properties)	87 untrained panelists	Greis et al. [78]
Plant-based alternatives to yogurt (including dairy control)	Consumer Acceptability (hedonic scales)	25 untrained panelists	Grasso et al. [85]
Plant-based alternatives to yogurt	Consumer Acceptability (hedonic scales)	15 untrained panelists	Huang et al. [82]
Plant-based alternatives to cheese (two trials= raw and melted)	Consumer Acceptability (hedonic scales, CATA, emotional response)	100 untrained panelists and 93 untrained panelists	Falkeisen et al. [96]
Plant-based alternatives to cheese	Acceptance Test	10 trained panelists	Li et al. [87]
Plant-based alternatives to cheese	Consumer Acceptability (hedonic scales)	50 untrained panelists	Arise et al. [92]

Although sensory testing typically focuses only on sensory properties, for PBAs, it may be important to also include an examination of the impact of extrinsic cues on the acceptability [28,126]. Extrinsic cues, such as the ingredient list, can influence a consumer’s liking of a product. For instance, peas or lentils could be disliked by consumers as they do not think they are a good meat substitute. PBAs also have long ingredient lists; this can lead to negative expectations for the consumption of the product and can reduce the willingness to purchase [127]. Consumers are concerned with the perceived “healthiness” and “naturalness” of food products and PBAs are often perceived by consumers to be unnatural and highly processed [27,128]. This attitude can lead to negative perceptions of PBAs.

The healthiness of food items and an unacceptable taste are correlated in consumers’ minds, and as PBAs are perceived to be healthy, this can also lead to negative experiences [80,119]. Studies need to investigate whether this is also true for the healthiness and texture perception of PBAs. Furthermore, price can also impact the sensory perception of food products, and as PBAs are usually more expensive than their conventional counterparts, this could be a barrier to their consumption [27]. All of these extrinsic cues contribute to the consumers’ conceptualism of PBAs [129]. Conceptualism is defined as the meaning or feeling attributed to the sensory and packaging experience of products [121]. Studies need to further explore how consumers’ conceptualism impacts their textural perception of PBAs. Furthermore, future studies should measure consumers’ emotional responses to PBAs in conjunction with their sensory perception. Emotional responses have been found to be able to differentiate products beyond hedonic measurements [130]. A study investigating PBCs asked consumers to evaluate both their emotional responses and the products’ sensory properties [95], and found that positive emotions were associated with liking. However, the study did not include extrinsic cues. Future studies involving PBAs should evaluate how different information (packaging, sustainability factors, nutritional claims) influences a product’s acceptability. Furthermore, most studies on PBMAs had them being consumed in isolation, without the usual condiments and additional ingredients (ex. burgers without condiments and buns). To achieve a more realistic sensory perception from consumers, these factors need to be included in sensory trials. In addition, the meal that the PBA is going to be used in affects the acceptability, and as stated by Elzerman [125], more studies are needed to investigate the use of PBAs in different meal combinations.

While understanding consumer acceptability is necessary for the successful uptake of PBAs, a methodology that characterizes product breakdown will provide a greater depth of information about the eating experience of a PBA [112,113]. Temporal Dominance of Sensations (TDS) is one such methodology. This approach studies the dominant sensations of a food item perceived during a certain time period of mastication [131], and it has been successfully used to evaluate plant-based and dairy-based yogurts [75]. The PBYs were found to have more variation in the mouthfeel after the beginning of mastication than the dairy yogurts, and the researchers hypothesized that this could be the result of a mixture of saliva and enzymes during oral processing. This demonstrates that dynamic sensory methods should be used to determine the complete textural profile of PBAs and these factors should be taken into consideration during product development. In addition to TDS, temporal check-all-that-apply (TCATA) could be used to evaluate the dynamic perception of PBAs. TCATA allows the participants to indicate all of the properties that they perceive simultaneously [132], and this could also be used to evaluate textural properties during the mastication process. Most sensory studies have asked the participants about the acceptance of the PBAs, but for them to be successful, many components need to be considered, including characterizing the textural properties (using both static and dynamic methods), clear objectives, and the consideration of the conceptualism of the products, as well as different consumer segments.

The development of new PBAs needs to involve sensory evaluation methods as consumer perception directly impacts the acceptability and purchase of PBAs. Future studies should continue to use instrumental measurements, but should pair them with sensory evaluation methods. Future studies should state whether the PBA is meant to mimic a conventional product and identify who the target consumer is, whether meat and dairy avoiders or meat and dairy consumers. Additionally, as new plant proteins are investigated, the textural properties, from the initial evaluation (looking at the product) through to the swallowing of the PBA, need to be investigated. PBAs are a growing area of research and a growing market segment in the food industry; sensory evaluation methods must be used to allow product developers to create more acceptable products and improve the PBAs that are currently being sold.

## 4. Conclusions

PBAs are a growing area of food production, and sensory science can complement other areas of scientific research. Plant-based products are difficult to produce as there are many different consumer segments and avenues for production (mimic conventional products or unique products); however, regardless of the type of product that is envisioned by the producer, PBAs may be linked to conventional products by the consumer. The drivers and barriers to the consumption of PBAs need to be further investigated and the sensory properties need to be improved. If PBAs are meant to mimic conventional products, then the textural properties need to more closely resemble those of the conventional products, as familiarity with the textural properties of conventional products will continue to hinder the consumer experience and consumption of PBAs. In addition, consumer segments need to be identified in future studies so that researchers can improve the design of PBAs to meet the needs and expectations of different consumers and consumer groups. Overall, sensory science researchers need to move beyond consumer acceptability trials or create more robust acceptability trials (ex. more consumers, attribute diagnostics, blinded and informed conditions, segmentation) to continue to improve PBAs. The existing studies discuss the importance of textural properties, and future sensory studies need to use dynamic methodologies to continue to improve the textural properties of PBAs. In addition, PBAs are associated with many extrinsic factors (e.g., sustainability), and these factors should be included in sensory trials to better understand the consumer and how these extrinsic factors interact with the sensory properties. It is necessary for detailed sensory evaluation studies to be completed to guide successful PBA product development.

## Figures and Tables

**Table 1:** Examples of the different studies investigating the textural properties of plant-based alternatives.

Type of Plant-Based Alternative	Method of Evaluation	Reference
Plant-based analogues to burgers	Texture Profile Analysis	Vu et al. [5]
Texturized Vegetable Protein compared to beef, pork, chicken	Water Absorption Capacity Integrity Index Texture Profile Analysis Cutting Strength	Samard and Ryu [33]
Plant-based analogues to nuggets	Texture Profile Analysis Consumer Acceptability (hedonic scales)	Yuliarti et al. [34]
Plant-based analogues to meat	Water Holding Capacity	Cornet et al. [35]
Plant-based analogues to burgers	Water Holding Capacity Texture Profile Analysis	Zhou et al. [36]
Plant-based analogues to sausages	Texture Analysis Consumer Acceptability (hedonic scales and JAR scales)	Dreher et al. [37]
Plant-based alternatives to yogurt	Texture Analysis Water-holding capacity Sorting	Canon et al. [38]
Plant-based alternatives to yogurt beverages	Texture Analysis Quantitative Descriptive Analysis	Mefleh et al. [39]
Plant-based alternatives to yogurt	Oscillatory Measurement Apparent Viscosity Consumer Acceptability (hedonic scales)	Pachekrepapol et al. [40]
Plant-based alternatives to yogurt	Water Holding Capacity Texture Analysis Rotary Rheometer	Xu et al. [41]
Plant-based alternatives to cheese	Texture Profile Analysis Cheese Stretchability	Mattice and Marangoni [42]
Plant-based alternatives to ice cream	Trained Panel Texture Profile Analysis	Pontonio et al. [43]

## Data Availability

Not applicable.
